# Climbing crags recommender system in Arco, Italy: a comparative study

**DOI:** 10.3389/fdata.2023.1214029

**Published:** 2023-10-11

**Authors:** Iustina Ivanova, Mike Wald

**Affiliations:** ^1^Independent Researcher, Bolzano, Italy; ^2^Department of Electronics and Computer Science, University of Southampton, Southampton, United Kingdom

**Keywords:** recommender systems, sport climbing, ranking, recommendations, preferences elicitation, recommender evaluation, user study, predictive models

## Abstract

Outdoor sport climbing is popular in Northern Italy due to its vast amount of rock climbing places (such as crags). New climbing crags appear yearly, creating an information overload problem for tourists who plan their sport climbing vacation. Recommender systems partly addressed this issue by suggesting climbing crags according to the most visited places or the number of suitable climbing routes. Unfortunately, these methods do not consider contextual information. However, in sport climbing, as in other outdoor activities, the possibility of visiting certain places depends on several contextual factors, for instance, a suitable season (winter/summer), parking space availability if traveling with a car, or the possibility of climbing with children if traveling with children. To address this limitation, we collected and analyzed the crag visits in Arco (Italy) from an online guidebook. We found that climbing contextual information, similar to users' content preferences, can be modeled by a correlation between recorded visits and crags features. Based on that, we developed and evaluated a novel context-aware climbing crags recommender system Visit & Climb, which consists of three stages as follows: (1) contextual information and content tastes are learned automatically from the users' logs by computing correlation between users' visits and crags' features; (2) those learned tastes are further made adjustable in a preference elicitation web interface; (3) the user receives recommendations on the map according to the number of visits made by a climber with similar learned tastes. To measure the quality of this system, we performed an offline evaluation (where we calculated Mean Average Precision, Recall, and Normalized Discounted Cumulative Gain for top-N), a formative study, and an online evaluation (in a within-subject design with experienced outdoor climbers *N* = 40, who tried three similar systems including Visit & Climb). Offline tests showed that the proposed system suggests crags to climbers accurately as the other classical models for top-N recommendations. Meanwhile, online tests indicated that the system provides a significantly higher level of information sufficiency than other systems in this domain. The overall results demonstrated that the developed system provides recommendations according to the users' requirements, and incorporating contextual information and crag characteristics into the climbing recommender system leads to increased information sufficiency caused by transparency, which improves satisfaction and use intention.

## 1. Introduction

Outdoor sport climbing (OSC) is a popular recreational activity in Northern Italy due to its vast amount of rock climbing places (Bollati et al., 2014). Typically, people climb on the natural rocks called crags, and their immense amount within Italy attracts many foreign tourists, practicing sport climbing in this country (Seifert et al., 2016). This increased interest from the population leads to a rise in the development of new climbing routes and crags and creates an information overload problem for people who plan their climbing trips. Recommender systems (RSs) are introduced as a tool to deal with such problems (Ricci et al., 2022) and have been intensively employed in other outdoor sports, e.g., running (Feely et al., 2022; Vardhan et al., 2022) and hiking (Posti et al., 2014; Calbimonte et al., 2020, 2021). In the OSC domain, a climbing recommender system (RS) provides crag recommendations based on the number of suitable climbing routes located within the crag (Ivanova et al., 2022b). However, this system did not consider climbing contextual information (season, transportation, and company) or crag-specific features (amount of routes within, length of the rocks, etc.). Very often, climbers plan their visits according to some outdoor-specific contexts: company with whom they travel (with family and kids/friends), how they travel (by bike/car), season and daytime when they plan their trip (e.g., winter/summer, morning/afternoon), and possible weather conditions (rain/sun). Those aspects influence climbers' choices, and to learn contextual factors' importance for the climbers' selections, various techniques have been developed by Context-Aware recommender systems (CARSs) (Adomavicius et al., 2022). One of the standard methods is to infer the context using data mining methods from implicit feedback, e.g., the number of user visits to a specified place (Jannach et al., 2018). Those techniques have been partially explored in a recent system (Ivanova and Wald, 2023), which allows to adjust the user's contextual preferences. However, this system needs a complete study (formative, online, and offline) with its stakeholders (outdoor climbers).

In addition to the problem of contextuality, there is a limitation with the current interfaces of existing climbing crag recommender systems. The latest user study showed that the interface of current websites for crag recommendations mostly lacks the adaptation to the users' needs (Helle and Takala, 2020). For instance, such aspect of the RS interface as preference elicitation (PE) for expressing users' tastes for specific attributes positively influences user satisfaction and affects the RS quality (Knijnenburg and Willemsen, 2009; Brusilovsky et al., 2020). PE *via* attribute weights originates from the field of decision analysis (Knijnenburg and Willemsen, 2015) and can be provided *via* an interactive interface where users can make alternations for their preferences (Tintarev and Masthoff, 2022). In the domain of sport climbing, several existing systems adopted preference elicitation interfaces that allow users to adjust some aspects (for instance, crags' location, climbing grades, and sun position) (Ivanova et al., 2022b), 27crags.com,^1^ ukclimbing.com.^2^ However, they limit users to express other important crag characteristics such as equipment for safety, rock types, walking distance from parking, amount of routes, and some users' contextual factors, e.g., the possibility of visiting it with children.

To address the outlined limitations, in this study, we hypothesized that OSC contextual information, similar to content preferences, can be modeled from climbers' feedback in the form of their logs from their electronic diary (e.g., climbing website 8a.nu).^3^ To test this hypothesis, we collected 793 climbers' logs for 107 climbing crags from Arco, Italy, during 14 years (2008–2021) and compared their recorded visits with crag characteristics. This analysis showed that some features correlate highly with user visits, meaning that the Pearson correlation coefficient can model contextual factors. Based on these results, we developed and evaluated a novel content-based CARS for outdoor climbing crags called Visit & Climb (V&C). In this system, we assume that users with similar preferences for crags' content and similar defined contexts would visit similar places. Thus, a sorted list of recommended items is provided according to the previous visits made by the most similar user. Then, the developed system is evaluated in three scenarios, namely, offline, formative study, and online. In the offline evaluation, the system predicts climbers' visits to places, and those results are then compared with the other regression models computed from the crags' features. We evaluated these models with the most common metrics for ranking prediction: Hit Ratio (HR), Normalized Discounted Cumulative Gain (NDCG), and Mean Reciprocal Rank (MRR) for the top-K recommendations (Gunawardana et al., 2022). Furthermore, for online evaluation, we developed an interactive web-based interface where climbing contextual factors and content preference weights can be adjusted by climbers *via* a PE visualization panel with sliders. The formative study includes three strategies of how outdoor sportspeople of different levels use web searches to find the potentially interesting crag for their trip. Moreover, the online user study is, then, performed in a within-subject design: 40 outdoor sport climbers were asked to find climbing crags for their hypothetically planned climbing trip in Arco. We gave participants three existing websites in this domain: 27crags.com, a content-based climbing RS (Ivanova et al., 2022b), and the system Visit & Climb. In this study, we measured several evaluation metrics using a trust model from the study mentioned in the reference (Pu et al., 2011). The overall results of offline RS evaluation showed that contextual information, similar to content preferences, can be modeled based on Pearson correlation and can be employed for the prediction of crags choices with high accuracy. The online evaluation showed that V&C provides a higher level of Information Sufficiency. At the same time, the results suggested that one should combine this developed PE visualization panel in the form of sliders with buttons to provide a more user-friendly design.

Overall, this study aims to answer two research questions (RQ) as follows:

**RQ1:** How to model climbers' preferences, including their contextual information, in the outdoor climbing crags recommender system?

**RQ2:** How to develop the preference elicitation interface for the crags recommender system so that users can express their contextual information and crags content choice?

## 2. Related work

The climbing recommender system is already a well-established domain: for instance, Wilkes and Janowicz (2008) developed an RS application for outdoor climbing routes, where a collaborative filtering method was employed to predict suitable routes. One of the main limitations of this developed solution is that the user should explicitly provide his likes, which is not convenient. Furthermore, it did not consider crags as recommended items. Ginantra et al. (2020) developed an additional RS to suggest mountains to climbing tourists based on the user profiles, where they modeled mountain peaks based on their content (such as beauty, altitude, location, cost, and pathway) and gave recommendations according to the content-based approach. Unfortunately, such a system again requires manual user input to understand their preferences, and it needs to consider contextual factors in its recommending algorithm. Some state-of-the-art RSs have been developed to automatically profile climbers based on electronic climbing guidebooks, which people use to record information online about their climbed routes. For instance, Scarff (2020) scraped the data from e-guidebook mountainproject^4^ and predicted the rating of climbing routes based on the past ratings given to the other items by a user. The main limitation of this system is that this model suggests only routes without considering crags as unique recommended items. Furthermore, it still needs to model the contextual information of a user and content knowledge of the place. A recent RS was developed by Ivanova et al. (2022a), who employed climbers' comments for crags recommendations based on the collaborative filtering approach: the suggestions are supplied as a ranked list based on the number of routes within the crag similar to those liked by the user in the past. Such a system learns users' tastes for route features from their e-logs and offers an interactive PE interface to adjust those. However, it lacks the adaptation to the contextual preference adjustments and does not offer context-aware recommendations. At the same time, the latest user study performed with climbers by Ivanova et al. (2022b) showed that some crags' characteristics significantly affect users' choices of potentially interesting crags. For example, such information as sun orientation toward the crag informs climbers to avoid visiting a sunny crag in summer because it can become too hot for climbing there. This issue was partly solved in the novel CARS by Ivanova and Wald (2023), describing the main advantage of contextual preference elicitation. However, this system lacks a detailed assessment (formative study and online and offline evaluation analysis).

In other similar outdoor-related sports, e.g., hiking or running, RSs have already addressed contextual information. For instance, Knoch et al. (2012) developed a context-aware and content-based approach for running route suggestions considering the pre-filtering method. The main limitation of such a system is that it cannot automatically learn the contextual factors of the runners, hence it requires their manual input. Additional CARS for running route generation was developed by Long et al. (2017), who computed contextual factors of the user by mining his current location and further adjusted recommendations according to the weather condition in this location. Unfortunately, this system does not allow runners to adapt their contextual factors manually, and receiving suitable recommendations for different areas can be more complex than the users' one. In another sport, such as hiking, an RS was developed by Posti et al. (2014), to provide hiking path suggestions considering the location of the other hikers. First, this system automatically measures the area of a target hiker; second, it searches for other people in potentially interesting hiking paths; and third, it suggests less crowded routes. The main limitation of this system is that some outdoor-specific contextual factors need to be addressed as well, e.g., seasonality and company of a user.

In the climbing domain, some of the contextual factors can be adjusted by the user while they search crags locations. For instance, the website ukclimbing.comand (27crags.com) offers users to choose their context with the PE interface. However, their contextual preference adjustments are somewhat limited by several factors, such as the distance between the items and weather conditions. In fact, several user studies were performed with outdoor climbers about their perceived views toward the interface of e-guidebooks: Lean (2010) pointed out that some significant usability issues should be solved, and Helle and Takala (2020) described common problems experienced by the users while they were searching for suitable crags with the existing system of 27crags.com. Those results have not been considered to improve the usability level of current climbing crags RS interfaces. Unfortunately, there are no standard rules for how the interface should be developed for e-guidebooks: they vary significantly in how they present their information about routes and crags, and this information discrepancy often confuses users.

To sum up, the described method in this study aims to overcome several limitations outlined in the literature review. First, the proposed RS extends the existing climbing crags RS by introducing climbing contextual factors and crags content preferences and then offers to learn those automatically from users' training e-logs. Second, climbers can change those computed preferences with the PE panel interface. Third, this system suggests climbing crags to the user according to the most similar climber in terms of her calculated selections.

## 3. Climbing crags visits prediction

**Figure 1:**
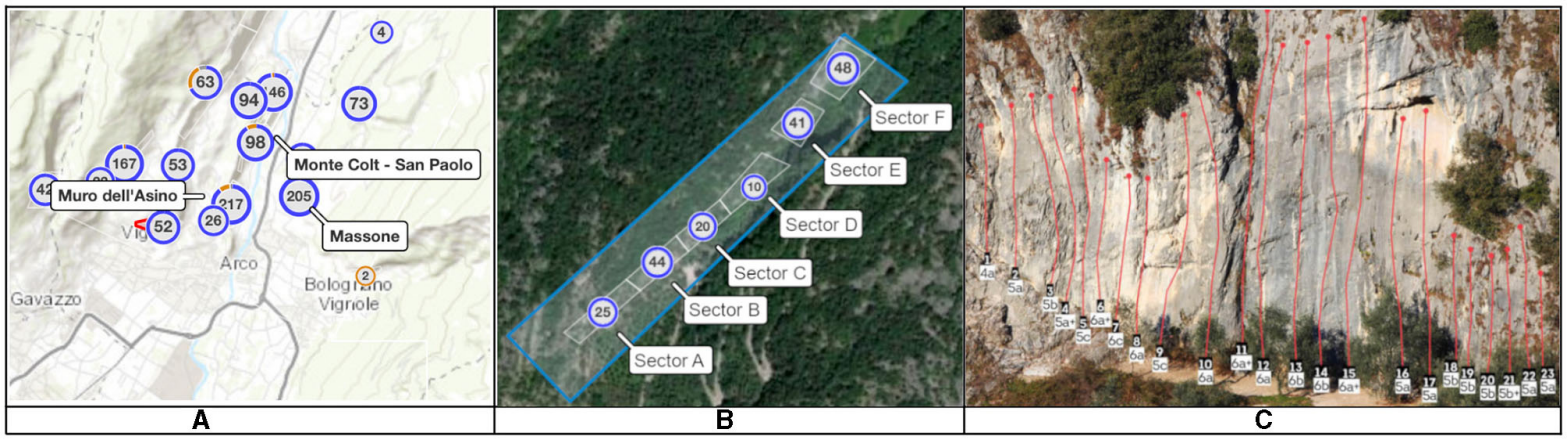
Schema of crags and sectors on the map from Arco, Italy: the existing interface of web climbing guidebook of crags and their granularity levels: **(A)** Map view of several crags (with the information about climbing routes located inside the blue circles)^5^; **(B)** Six climbing sectors located in the crag Massone^6^ (the number is related to the number of routes within); **(C)** One of the sectors from Massone with climbing routes, where the numbers and letters in white boxes show routes' grades and the red colored lines indicate position of the path on the rock wall (from Vertical-Life mobile application^7^).

Climbing crags consist of one or several natural rocks, where experienced climbers (route setters) install bolts so that every ascending sportsperson can place their equipment (e.g., quickdraws or carabiners) for their safety. Moreover, rock lines equipped with those bolts form climbing routes. Typically, those routes are not higher than 50m, and a safety chain is installed at the end of the way to help climbers abseil down. The e-guidebook usually shows sport climbing crags as points on a map or items in a table view. The schema of a map with several crags from Arco (Italy) is shown in Figure 1A: blue color indicates the location of the crag, and the number inside the circles shows how many crags are located in this circle. Furthermore, a climbing crag might consist of several sectors within walking distance. For instance, six sectors from crag Massone are shown on the mapping schema in Figure 1B: the number in this case indicates the number of climbing routes within walking distance. Moreover, a climbing sector has one or more climbing routes: Figure 1C shows a schema for one of the sectors from Massone crag with 23 climbing routes. In addition, climbing routes have different difficulty levels, for instance, 5a, 6b+, and 7a. This study adopted the French grade system, where numbers, letters, and the ‘+' symbol are used (Draper et al., 2015; Draper, 2016).

Usually, in a climbing e-guidebook, crags' information consists of their characteristics (features) defined by the route setters or people who commented on guidebooks online. In this study, we provide 33 crags' attributes and their descriptions from the electronic guide “Vertical-Life.” Those features form climbing contextual factors and climbing crag content-related information; some are binary variables (“Sun exposure,” “Rain safe,” “Family-friendly,” “Overhang,” “Roof,” “Vertical,” and “Slab”), while others are categorical (“Parking,” “Approach Time,” “The number of climbing routes,” “Wall Steepness,” “Safety,” “Shortest Route,” “Longest Route,” and “Rating”). We also use icons to visualize crag-related information in the system accordingly (see Table 1 for all features' descriptions, icons, and possible values).

**Table 1:** Climbing crag features and their relation to contextual information and content of crags (^∗^Icons are taken from Vertical-Life climbing application).

**Feature group**	**Feature name**	**^∗^Icon**	**Possible values**	**Explanation**
**Climbing contextual factors**	Sun exposure: “North,” “East,” “South,” and “West”	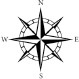	To which cardinal point crag is exposed toward the sun	Sun position toward the crag
	Parking	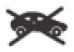	No parking	Parking spot availability. This attribute is important in a country, such as Italy, since many crags can be reached by car only. At the same time, the roads are too narrow to park a car; thus, this information is crucial for tourists' decisions.
			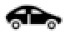	Bad parking	
			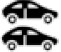	Good parking	
			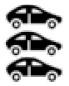	Excellent parking	
	Rain safe	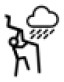	Protected against rain	Indication whether climbing is possible during rain
			-	Not protected against rain	
	Family-friendly	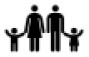	Family-friendly	Indication whether it is suitable for climbing with children
			-	Not Family-Friendly	
**Climbing crags' content information**	Approach time (minutes)	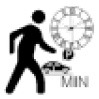	1, 2, 3, 5, 8, 10, 12, 15, 18, 20, 25, 30, 40, 45	How much time does it take to walk from the nearest parking spot to the crag?
	The number of climbing routes for 14 grades/difficulty levels: <5, 5a, 5b, 5c, 6a, 6b, 6c, 7a, 7b, 7c, 8a, 8b, 8c, >8c	-	0, 1, 2, 3, 4, 5, 6, 7, 8, 9, 10, 11, 12, 13, 14, 15, 16, 17, 18, 19, 20, 21, 22, 23, 24, 25, 26, 29, 31, 33	It shows how many routes of specified climbing difficulty levels are present within the crag.
	Wall Steepness	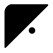	Overhang	The rotation angle of the wall/type of wall steepness in the crag. For Overhang, this angle is between 95° and 165°, for Roof this angle is > 165°, for Vertical this angle is between 88° and 95°, for Slab this angle is <88°.
			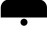	Roof	
			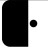	Vertical	
			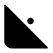	Slab	
	Type of rock	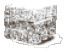	Porphyry	Type of rocks. There are more rock types in general, but there are two that exist in Arco.
			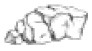	Limestone	
	Safety	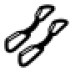	Safe	Safety level of the crag: how far away bolts are located from each other on the wall
		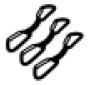	Very safe	
	Shortest route	-	4, 5, 6, 7, 8, 9, 10, 12, 13, 14, 15, 16, 17, 18, 20, 22, 25	The minimum height in this crag
	Longest route	-	10, 11, 12, 14, 15, 16, 18, 20, 22, 23, 24, 25, 26, 27, 28, 30, 31, 32, 33, 34, 35, 36, 38, 40, 41, 42, 45, 50	The maximum height in this crag
	Rating	-	1, 2, 3, 4, 5	Crags' rating in guidebook

As has been found out, some crags' features significantly affect sport climbers' choices of visits: for instance, the component of “*Number of climbing routes”* suitable for the current climber's level is crucial for a person, as she cannot visit crags where routes' difficulty is above their physical capability. Furthermore, for some sportsmen, a feature such as “*Type of rock”* matters more: they prefer limestone over porphyry, as the last is characterized by evident cracks and vertical plates, which requires a particular type of preparation (De Giorgi et al., 2021).

To learn what a climber prefers, we hypothesized that the user's visits correlate with the climber's preferences because the climber would not visit some crags unless she liked something about it. To check this hypothesis, we collected 793 climbers' logbook data recorded for 128 climbing crags on the webpage of www.8a.nu^8^ within Arco, Italy. In addition, we collected information about places with the features described above. Some anonymized information is publicly available online in GitHub (Ivanova, 2023).

**Figure 2:**
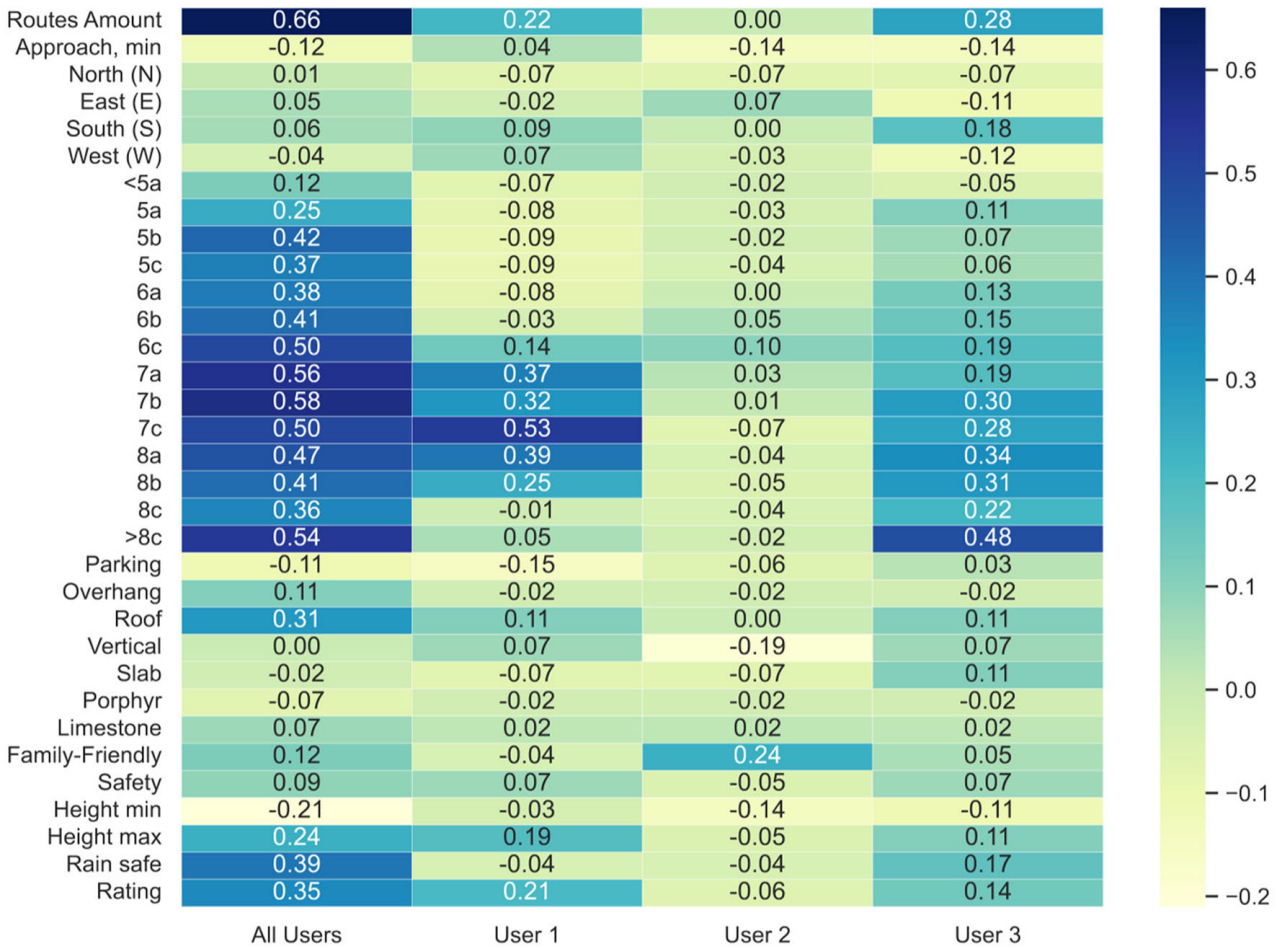
Correlation of heat map for crags' visits and features. X-axis shows for whom the correlation is measured (from left to right): 1st column–for ***All users***, 2nd column–for ***User 1***, 3d column–for ***User 2***, 4th column–for ***User 3***, Y-axis shows related features.

To answer RQ1, we analyzed whether there is a correlation between certain features and amount of visits. For example, we plot a heat map showing this correlation for several interesting cases from the data in Figure 2. In this figure, the first left column shows the correlation for **“*All users”*** (where we included 793 climbers), and the second, third, and fourth columns show correlations for three existing climbers from the database, namely, **“*User 1”***, **“*User 2”***, and **“*User 3”***, accordingly. The blue color illustrates a positive correlation, and the white-yellow color is negative. We can observe from the first column that the feature of “*Routes amount”* is positively correlated (0.66) with the number of visits for **“*All users.”*** Moreover, the more amount of “*7a”*, “*7b”*, and “>*8c”* grades related to the increased amount of all climbers' visits (correlations for specified features are 0.58, 0.58, and 0.54, accordingly). Furthermore, one can conclude from the second column of the figure that **“*User 1”*** is more interested in visiting crags with a larger value of “*7c”* grades (his visits' correlation with this feature is 0.53). In contrast, **“*User 2”*** (column three) has the highest correlation (0.24) with the “*Family-friendly”* feature (probably, he is traveling with family and children, thus can only visit crags suitable for children). Finally, **“*User 3”*** (fourth column) is more interested in climbing “*>8c”* grade routes (correlation with this feature is 0.48). We can conclude that there is a clear correlation between certain crag features and the number of user visits; thus, we propose to model the subjective preferences based on this conclusion. To do so, we employed the Pearson correlation for users' profiles. We used the following equation to model the tastes of a user *u* for each feature *f* :


(1) $$P_{u_{t}f_{c}}=\frac{\sum_{c=1}^{N}\left(f_{c}-\bar{f_{c}}\right)\left(v_{u_{t}c}-\bar{v_{u_{t}}}\right)}{\sqrt{\sum_{c=1}^{N}{\left(f_{c}-\bar{f_{c}}\right)}^{2}}\sqrt{\sum_{c=1}^{N}{\left(v_{u_{t}c}-\bar{v_{u_{t}}}\right)}^{2}}}$$

where *f*_*c*_ is a specified feature of a crag *c, N* is the overall amount of crags, $\bar{f_{c}}$ is the average value of feature *f*_*c*_ among all crags, *v*_*u*_*t*_*c*_ is the number of visits made by the user *u*_*t*_ to crag *c*, $\bar{v_{u_{t}}}$ is the average amount of visits made by the user *u*_*t*_ to all crags; and **u_t_** is vector representation of preferences of a target user. In this formula, the correlation produces the relevant score toward the importance even for binary features, as it normalizes their values with a summation.

For computing similarities, we used another Formula 2: it shows the most similar user from all climbers (for similarity computation, we included 654 climbers with at least 5 unique crags recorded in their logs).


(2) $$k=\underset{u_{k}\in U}{\arg\;\min}(dist(u_{t},u_{k}))$$

where *k* is the most similar user id, **u_k_** indicates preferences vector of user *k*, **U** presents set of all users; *dist*(**u_t_**, **u_k_**) computes distance between two users.

## 4. Experimental study

To answer the research questions posed in this study, we conducted three studies: the first one was the offline evaluation of several recommender models for ranking prediction; the second one was a formative study where we interviewed climbers about their view on climbing crag RS; and the last one was an evaluation of the developed system V&C. Based on the findings from those studies, we defined several goals to overcome the limitation of current systems, providing climbing crag search and recommendations.

### 4.1. Offline evaluation

We employed two formulas given above for user taste modeling and developed novel CARS for crags called Visit & Climb. It consists of three steps as follows: (1) contextual information and content tastes are learned automatically from the users' logs by computing the correlation between users' visits and crags' features (Formula 1); (2) PE interface described in Section 4 provides weights for interactive slider tool adjustments, the user adjusts his tastes; (3) recommendations are forecasted as the previous visits made by the most similar user (vector of preferences weights for existing users is computed from their logs based on Formula 2). The third step follows the collaborative filtering approach and assumes that similar climbers would visit similar crags. For the purpose of similarity computation, we compared several metrics and chose the best-performing one based on its accurate suggestions (distance metric = “dice,” algorithm = “ball_tree”).

For the offline evaluation scenario, we selected 106 users who visited at least 20 unique locations, and 99 items reported being visited by at least 3 climbers. Then, we created a user-item interaction matrix for each user, where each cell represents the number of visits a particular climber made to a specific crag. If the user recorded several routes within the same place for a particular day, we consider this one visit; thus, we assume only unique crags' visits per day. To avoid possible bias connected with the fact that many climbers visit the same place many times because of their living location (it might be that they live very close to this place), we made unique crag visits limited to three amounts (we included only crags of first two records and the last one, per user). This idea comes from the fact that climbers typically use recommendations while they travel to a new destination. Since traveling time is limited, they only visit the same place for up to 3 unique days. Furthermore, crags' ratings are measured as average ratings given by the other users for climbing routes within this crag (as provided in the e-guidebook).

We split the data by time: for each user, we split the data in a ratio of 80 and 20% (the earliest 80% of the ascents were used for training, while the remaining logs were for testing purposes). As a baseline model for evaluation, we assumed predicted values in the testing set to be the same as in the training set. To further evaluate and compare the V&C performance, we considered the evaluation of other top-N recommender models: several regression-based models for ranking forecasts, such as RandomForest,^9^ LinearRegression,^10^ and XGBoost.^11^ The main advantage of these systems is they learn users' preferences from their past likes and crags' features. In addition, we tested singular value decomposition (SVD)^12^: unlike the regression-based systems, and it does not require item feature information.

Furthermore, for each of the systems above, we performed cross-validation on a train data set (number of folds = 3), to find the best parameters of the models (hyperparameters tuning). Thus, in the resulting table, we report evaluating the parameters found during this process. For the evaluation of models, we measured the commonly reported metrics to the ranking assessment, such as Mean Average Precision (MAP@k), Recall (Recall@k), and Normalized Discounted Cumulative Gain (NDCG@k) at top-k (k = 1, 3). The values of the metrics vary from 0 to 1, with the worst ranking quality equal to 0, while the best ranking will be 1. The results of the models' pre-training are presented in Table 2. This table shows that the V&C provides significantly better accuracy for all metrics than the baseline. Even though item-based KNN, XGBoost, and RandomForest have higher accuracy than the V&C, their results are statistically not better in all the cases (for this purpose, we employed the *t*-test). We assume that more users in the comparison dataset can solve this issue since there are now only 654 users. Thus, we conclude that V&C performs comparatively accurately than the other recommender systems.

**Table 2:** Results of MAP@k, Recall@k, and NDCG@a for recommended list of crags.

	**Systems evaluation based on time-split per user**						
**Metrics**	**Baseline**	**Visit & Climb**	**XGBoost**	**RandomForest**	**Linear regr**.	**SVD**	**KNN (items)**
**MAP@1**	0.028 (±0.167)	0.377 (±0.487)^*^	0.396 (±0.496)^*^	0.33 (±0.473)^*^	0.264 (±0.443)^*^	0.406 (±0.493)^*^	**0.415 (±0.495)** ^*^
**Recall@1**	0.005 (±0.03)	0.053 (±0.073)^*^	0.057 (±0.075)^*^	0.048 (±0.072)^*^	0.039 (±0.068)^*^	0.056 (±0.074)^*^	**0.058 (±0.074)** ^*^
**NDCG@1**	0.018 (±0.089)	0.29 (±0.364)^*^	0.292 (±0.396)^*^	0.244 (±0.38)^*^	0.201 (±0.367)^*^	0.289 (±0.397)^*^	**0.294 (±0.396)** ^*^
**MAP@3**	0.112 (±0.211)	0.487 (±0.427)^*^	0.518 (±0.418)^*^	0.53 (±0.4)^*^	0.443 (±0.378)^*^	0.508 (±0.425)^*^	**0.531 (±0.424)** ^*^
**Recall@3**	0.054 (±0.103)	0.131 (±0.124)^*^	**0.162 (±0.145)** ^*^	0.159 (±0.135)^*^	0.145 (±0.126)^*^	0.139 (±0.127)^*^	0.144 (±0.125)^*^
**NDCG@3**	0.082 (±0.142)	0.269 (±0.251)^*^	0.308 (±0.291)^*^	**0.311 (±0.291)** ^*^	0.25 (±0.259)^*^	0.279 (±0.281)^*^	0.228 (±0.276)^*^

The best values for each test set and metric are in bold, the second-best models are denoted as underlined fonts. Statistical significance level with the baseline: ^*^p <0.0001.

We argue that the advantage of V&C is that it employs the learned tastes' weights in the interactive interface and overcomes the cold-start problem. One can oppose that they can similarly utilize the linear regression weights for the preference panel. However, in this case, the coefficients can not be normalized with Pearson correlation coefficients, which have minimum and maximum values of –1 and 1. Thus, presenting linear regression weights to the climber in a preference elicitation tool is tricky.

### 4.2. Formative study

To answer the second research question from Section 1, we collected reviews from 25 climbers (where we asked them to give us feedback in a written form about their thoughts on existing e-guidebooks, such as falesia.it^13^ and 27crags.com) and, in addition, 48 climbers regarding the existing content-based climbing RS (Ivanova et al., 2022b). Furthermore, we performed an exploration experiment about how experienced climbers searched for a climbing crag. For this purpose, we asked three climbers familiar with the problem of finding suitable rocks to find one for their possible planned vacation in Arco.

#### 4.2.1. Reviews received regarding crags' characteristics inclusion

For this step, the participants' selection was as follows: they had to be familiar with outdoor climbing, experienced the problem of searching crags, and used e-guidebooks before their climbing trip planning. We asked the participants to comment their view about climbing recommender for crags. The exact question we asked for this purpose is: “*Can you please add your comments about the climbing crag recommender system?”* After collecting the reviews, we analyzed them manually with the software developers and addressed the limitations according to the advices in the new version of the system.

Most participants reflected positively about climbing RS, while they pointed out that they experience problems with the existing e-guidebooks. Comment from a local (Arco) professional climber is as follows:

“*Add* the access, the sun exposition*, the morphological traits of climbing, the history of an ascent).”*

He suggested that “*approach time”* and “*sun exposure”* features are essential for the crags' choice.

Another climber gives a valuable comment as follows:

“*I would like a* more concise list of recommendations*. Also, would be nice to have information on* the best season */* time of the day *for the recommended crags.”*

We can observe that he was optimistic about the RS and pointed toward the quality of the list of recommended crags and the best season/time (features “*sun exposure”* and “*suitable month for climbing”* in Table 1).

One more interesting comment is received about the wall steepness of the crag as follows:

“*Give the location of the sectors relative to the* sun position*: if there is the shadow sector or not, as well as the angle of inclination (*wall steepness*) - a* positive wall, vertical*, or* overhanging*. I did not immediately find this information on my smartphone, and it would be convenient to have it on each route.”*

From this comment, we can conclude that the climbing travelers consider the aspects of shadow in the sector and angle inclination (“*sun exposure”* and “*wall steepness”* in Table 1).

#### 4.2.2. Reviews received regarding the PE interface of a RS

Regarding the interface of a system, we received the following comment:

“*To transform* grades scroller *instead of choosing the grades from check boxes.”*

This review was another reason to develop sliders for grades: a slider can indicate how many routes a user would like to see within desired grades.

Another person suggested using sliders for choosing grades as well:

“*Climbing grades - right now, we can choose only one category, for example, 7a–7a+. It is absolutely uncomfortable because, for example, I might want to climb routes from 5a to 6a+ inclusive for warm-up. Better take another type of control such as a* slider with two sliders *(as in moon application) and immediately select categories in the range from and before.”*

#### 4.2.3. Exploration experiment how users search climbing crags

During this experiment, we video-recorded how three experienced outdoor climbers searched for the crags for their planned trip to Arco. This study aimed to understand how people search for crags with web tools. Hence, we selected participants familiar with web e-guidebooks, had at least 1 year of outdoor climbing experience, and had traveled before practicing outdoor climbing for their vacations. We also wanted to analyze the search strategy for several different groups of climbers based on their level. Therefore, following the unified approach of climbers classification (Draper et al., 2011, 2015), which is based on how many attempts a person made to climb the route of a specified category without any falls entirely, we divided the participants based on their highest grades made redpoint (redpoint means to ascend a climbing route of selected category without any falls). We have found three male participants with different levels: intermediate (who can be defined as 6c redpoint level), advanced (redpoint level is 7a+), and elite (redpoint level is 8a+). Since the experiment aims to be qualitative rather than quantitative, we considered only three people for this experiment.

The exact task was as follows: “Suppose you plan a climbing trip to Arco (Italy). You have several days of climbing and would like to find interesting climbing crags for this trip. You can plan this trip with your girlfriend/boyfriend, wife/husband, kids, etc. Perform your normal search procedure to find what you would potentially look for while trying to find a suitable climbing place to visit (30 min).” Each session lasted for 40 min: the first 30 min allowed participants to explore any web search tool they preferred, then the last 10 min were used for asking several questions regarding the Visit & Climb system interface. During the user exploration step, participants showed their standard efforts when searching interesting crags, and their activities showed what is more important for each person. During this session, we asked several questions: (1) What tools do you usually use to find climbing crags with the web tool? (2) What type of information source (other than web tools) will you also look into? (3) Which features of climbing crags are the most crucial for your decision?

Among the participants, the first person (intermediate level) was based in Russia: he searched through google,^14^ plus explored the website of https://27crags.com for this purpose. He pointed out that the grades are the most important aspects for him, as he would not visit a crag with higher levels than he can climb (feature “*The number of climbing routes for fourteen grades/difficulty levels”*). Moreover, he explained that “*Approach time”* and “*Sun exposure”* are additional crucial parameters that would lead him to choose the place. The second person (advanced level) was from the UK: he used the British electronic guidebook (ukclimbing.com): interestingly, while searching for the crags, he was looking for the **comments** of the other visitors rather than the search *via* interface and PE panel; therefore, he wanted to find good reviews for the place visited by the others. Plus, he was interested in high crags' ratings; he assumed that the place which is not popular might also not be well maintained by the route setters and, thus, not pleasant to climb, that is related to the “*Rating”* feature from Table 1. The third person (elite level) was from Bulgaria, and he first asked some friends through the social network for recommendations. Then, he used the website 8a.nuto to find more information, where he sorted the crags according to the desired grades; then, he read the comments and ratings given for the routes and the number of ascents for grades he was specifically interested in trying. His decision was based on overall ratings, ascents, and reviews.

We can conclude that different climbers' levels can be why they use different information to decide where to go; hence, one should consider different strategies for the climbers based on their preferred methods. The system V&C is developed to target the intermediate level's strategy and addresses their desired parameters to adjust. Plus, it provides ratings of crags, following the advanced level. As we can observe, it is not tailored to elite climbers.

As a result of this performed formative study, we outlined the design goals positioned for the design of a new RS of Visit & Climb as follows: (1) to develop a better tool for expressing climbers' preferences with sliders; (2) to provide a list of recommended crags based on the visits of the most similar climber; and (3) to further evaluate proposed system design and functionalities.

### 4.3. Recommender system interface

**Figure 3:**
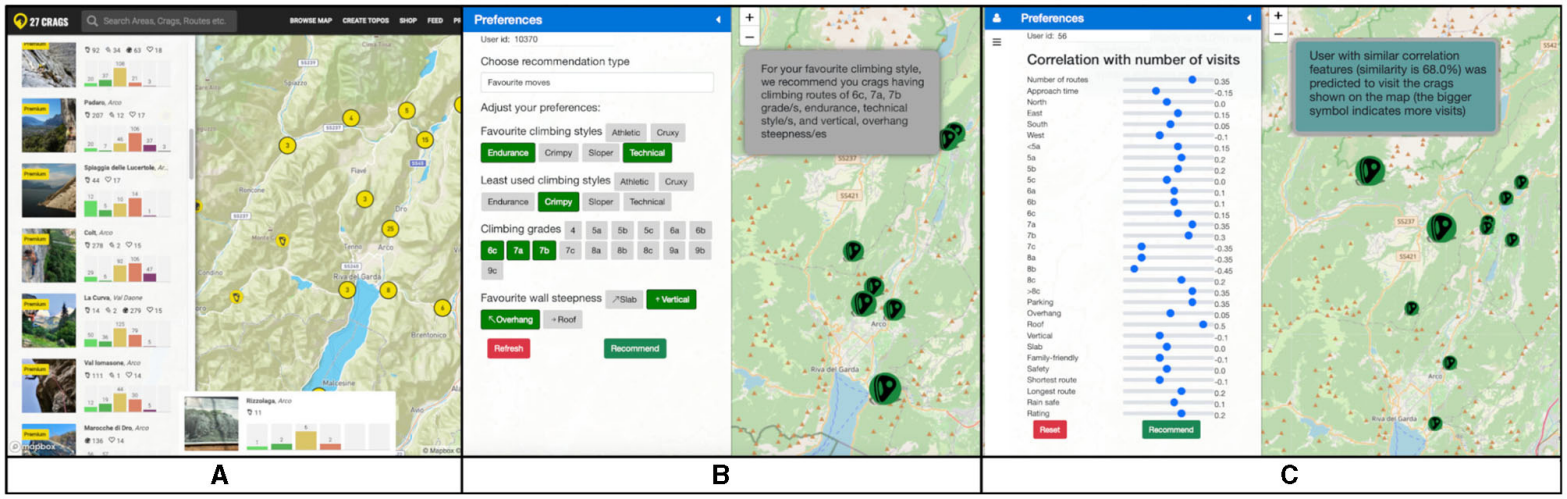
Three systems' interfaces were given to climbers to find crags in Arco, Italy: **(A)** www.27crags.com; **(B)** based on the content of the climbing routes (Content-Based RS) (Ivanova et al., 2022b); **(C)** based on the visits of the previous climbers (Visit & Climb) (Ivanova and Wald, 2023).

To address the design goals identified by the expert interviewers, we created an interactive climbing RS with a slider-based PE panel (the website is published online^15^). The system's interface is shown in Figure 3C. On the left side of this site, there is a PE panel for crags' features, which indicates the correlation between a feature value and the user's visits made (computed from Formula 1). The sliders' values vary from minimum correlation value for the target user to maximum correlation (for instance, the minimum is –0.5, and the maximum is 0.5 for the user with id = 56); if the value is negative, the feature is negatively correlated with the number of visits (meaning that the user is less likely to visit the crag if the feature value is higher), while positive correlation shows that the climber is more likely to choose the rock if the value is higher. The value closer to one leads to a larger correlation of a specific feature, and indicates a higher importance of this feature for a user; in contrast, the value closer to minus one leads to a negative correlation of a feature, and less level of it's impact. Suppose the user has not visited any crags yet, and he did not log any ascents in his diary (which is considered to be a cold-start problem). In that case, she should adopt the preferences manually by moving the slider to the left or right (the middle position indicates zero correlation, and this feature does not play a significant role in her decision). When the climber adjusts all the sliders according to what is more or less critical, he would see recommendations on the map (the crags are shown interactively with the circle symbol of a bolt), plus on the table as a suggested list of rocks. This ranked list is given according to the visits of the most similar user in terms of his preferences defined, and the higher value of recorded logs by this nearest user would also lead to a more giant symbol of a crag shown on the map. Moreover, on the website, there was an explanation sentence showing why this climber would see recommended crags, to which we included the distance to the nearest neighbor found for his preferences (see Figure 3C). An example of the sentence is given below:

*User with similar correlation features (similarity is 68.0%) was predicted to visit the crags shown on the map (the bigger symbol indicates more visits)*.

### 4.4. Recommender system online evaluation

It has been shown that an offline evaluation is insufficient for an RS, and that, a proper assessment of RSs requires conducting user experiments (Knijnenburg and Willemsen, 2015). For the described system, we performed a user study with experienced outdoor climbers (within-subjects design, *N* = 40). We asked them to find several crags for their planned climbing vacation to Arco, utilizing three existing systems. Figure 3 shows interface of these systems as follows: (1) 27crags.com, which was selected as one of the most frequently used by the other climbers for crag search: its interface supports users inserting certain constraints for crag features (see Figure 3A); (2) climbing crag RS based on the content of the climbing routes **Content-Based RS** (Ivanova et al., 2022a), which was developed based on the 27crags.com (Figure 3B), and the proposed system **Visit & Climb** (Ivanova and Wald, 2023) (Figure 3C). The systems use the same information about climbing crags, and they have similar map-based functionality to show crag information, with the difference in the interface of the PE panel and the recommendation algorithms. 27crags offers a PE panel based on filters to define climbing grades and provides the most visited crag recommendations based on the number of suitable climbing grades within. The second system (Content-Based RS) offers PE for routes' grades and rocks' styles and provides recommendations based on the number of suitable routes within the crag. The third system (Visit & Climb) offers PE for context and crag content and recommends based on collaborative filtering.

Participants were given the same assignments for each of the systems (in random order). The exact task was: “Consider a scenario when you (a climber) are planning to come to Italy (Arco) for a climbing vacation for 7–10 days (maybe with girlfriend/boyfriend, or family and kids). Your task is to find 2–3 climbing crags to visit during this vacation using the system.” While the participants interacted with the systems, we recorded their screens and video-tracked their activities on the website (mouse interaction). After the users tried out the websites, they were asked to answer the questionnaire, which measures the system's quality and recommended items and demographic questions. We adopted the user-centric evaluation framework by Pu et al. (2011) for RS evaluation. The RS questions measure several recommendation metrics: (Q1) Recommendation Accuracy, (Q2) Novelty, (Q3) Diversity, (Q4) Interface Adequacy, (Q5) Explanation, (Q6) Information Sufficiency, (Q7) Control, (Q8) Transparency, (Q9) Perceived Usefulness, (Q10) Overall Satisfaction, (Q11) Confidence and Trust, (Q12) Use intention, and (Q13) Purchase Intention.

We recruited participants through social networks and from the local climbing gym (Salewa-Cube, Bolzano^16^). Participants were from Italy, Russia, UK, Turkey, Moldova, Australia, Austria, Kazakhstan, Spain, Germany, Mexico, and Bulgaria. The mandatory selection was that the participants had to be experienced climbers: they all had to be familiar with reading web e-guidebooks (meaning that they knew how to search crags in a typical website guidebook), plus they had to climb outdoors several times, having more than 1 year experience of such. For this experiment, we did not classify climbers based on their levels because we were mainly interested in the overall population perception, not within the different climbing groups. There were 25 men and 15 women, and their ages ranged from 18 to 75 years, with the average age being 40 years old. By performing this experiment, we expected that the system Visit & Climb would be better than the baseline (www.27crags.com), and we wanted to understand in which aspects it would be perceived better than the content-based RS. Each question response was scaled between 1 and 5, where 1 presents completely disagree and 5 presents absolutely agree. The session lasted for at least 15 min of interaction with the system and 10 min of answering questions.

**Figure 4:**
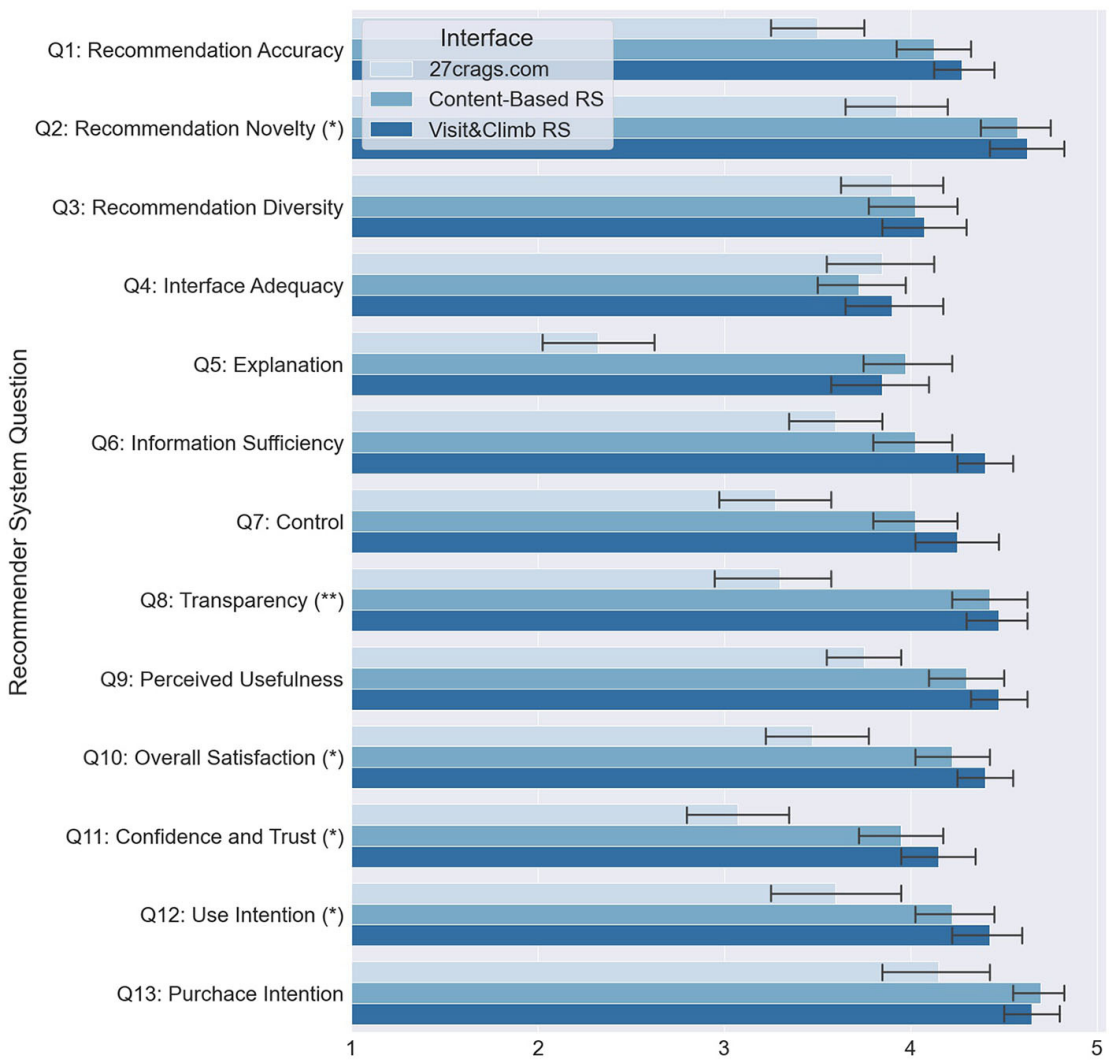
User feedback analysis: the results show that the Visit & Climb interface receives a significantly higher value in the aspect of Recommendation Accuracy (Q1), Recommendation Novelty (Q2), Explanation (Q5), Information Sufficiency (Q6), Control (Q7), Transparency (Q8), Perceived Usefulness (Q9), Overall Satisfaction (Q10), Confidence and Trust (Q11), Use Intention (Q12), and Purchase Intention (Q13) (Statistical significance level: ^**^p < 0.01; ^*^p < 0.05).

Then, we performed the ANOVA test to measure the significance level of the difference between the three systems in terms of RS evaluation metrics. In addition, we measured which systems among the three significantly vary for particular metrics with Tukey's honest significant difference test (Abdi and Williams, 2010). A total of 40 participants are considered to be a sufficient amount of users for this type of study (the systems were counterbalanced between participants). The results of those tests showed that V&C receives a significantly higher value than the system of www.27crags.com for Q1, Q2, Q5, Q6, Q7, Q8, Q9, Q10, Q11, Q12, and Q13. In addition to that, V&C outperforms also Content-Based RS significantly in Q6. These results are shown in Figure 4.

**Figure 5:**
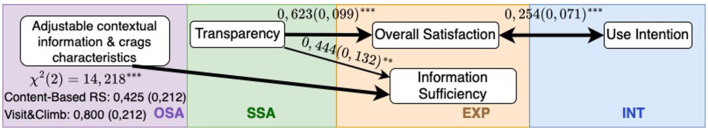
Graphical presentation of the path model. The number (thickness) on the arrows represents the β coefficients and standard error of the effect (in brackets). χ^2^ is a chi-square test performed for three systems (Statistical significance level: ^***^p < 0.001 and ^**^p < 0.005).

Furthermore, each question is related to one of the conceptual components from the evaluation framework of Knijnenburg et al. (2012) and Knijnenburg and Willemsen (2015): OSAs, Objective System Aspects; SSAs, Subjective System Aspects; EXP, User Experience; INT, Interaction. We ran a path model to understand why V&C improved information sufficiency significantly. With this analysis, we found out that this effect was caused by transparency. At the same time, transparency is also related to satisfaction, which is, in return, correlated with use intention. We can conclude that including context factors and crags' characteristics in V&C interface provides a higher level of transparency (e.g., users understand the item better with this additional info), which increases information sufficiency significantly and also affects satisfaction. The path model (Figure 5) has a reasonable model fit: CFI = 0.971, TLI = 0.958, the *p*-value for RMSEA <= 0.05 is 0.242.

### 4.5. Findings

Through the user study performed, we identified several vital points that the climbers commented on regarding the proposed interface of Visit&Climb system. These points are related to the several aspects of the system, namely, (1) PE visualization panel; (2) recommendation algorithm; (3) explanation sentence; and (4) crags' information. The second and fourth items answer RQ1, and the other address RQ2. We identified limitations through personal conversations with the participants which were audio-recorded; therefore, we summarize the crucial issues pointed by the participants (in a qualitative form).

1. **PE visualization panel:** Many climbers pointed out that adjusting too many sliders takes a lot of time; thus, one should develop fewer tools in PE. Thirty-three sliders are provided in the system now, which take much effort to adjust; hence, the new version with a few most important elements should be developed. For instance, one person suggested using “tags,” which would allow climbers to choose which features they prefer first to adjust. Moreover, people perceived sliders as a good visualization for grades (such features as <5, 5a, 5b, 5c, 6a, 6b, 6c, 7a, 7b, 7c, 8a, 8b, 8c, and >8c), but for other features, it would be better to use buttons/switch tools instead of sliders, since it is a more convenient way to express preferences for those. This suggestion is because climbing grade distribution is important for choosing crags. At the same time, such values as “North” and “Rain safe” are binary variables, which can be expressed differently, e.g., climbers either choose “North” when summer or not. Unfortunately, approximately 40% of the climbers we interviewed did not like the PE panel interface with the sliders; even though they liked the recommended items, they did not want to spend their time understanding the meaning of each slider.
2. **Recommendation algorithm:** Interestingly, many climbers found that the recommendations that have been provided by the Visit & Climb system satisfy them more than the recommendations given by 27crags.com and Content-Based RS. The system worked very well for lower grades [climbers whose redpoint level is less than 6a according to Draper et al. (2015)], intermediate (men: climbers who climb routes between 5c+ and 7a, women: between 5c+ and 6b+), and advanced (men: redpoint level is between 7a+ and 8a; women: redpoint level is between 6c and 7b+). Elite climbers targeting grades of more than 8a (men) have periodically received inaccurate recommendations. This case was because there were not enough users in the data who would climb at those levels (only 5% of climbers in the database could climb 8b-9a). Normally, a collaborative-based system that computes user similarities provides better recommendations if more users are involved. At the same time, even with 654 users, V&C gave accurate suggestions regarding the expected grades. Recommended items also suited the expected context for most cases when the sliders were adjusted properly. That means applying Formula 1 for climber profiling and Formula 2 computing similarities between the target user and existing climbers provides accurate recommendations and tailors to their expectations.
3. **Number of recommended crags:** RS system Visit & Climb sometimes delivered only five recommended crags because, in the respective data set of users, some visited only five crags in Arco. In this case, climbers have perceived only five recommended items as insufficient. Thus, more recommended items (at least 10) should be provided.
4. **Explanation sentence:** The explanation sentence was perceived as understandable by the climbers. Some users liked the fact that in the recommended crags, there have been several climbers already; thus, they better trusted this system. At the same time, some users found the explanation sentence to be too complex (for instance, the similarity level in percentages was suggested to be removed). Moreover, not every climber can observe the location of the box in the interface with the explanation sentence, so a clearer way to show the explanation should be developed. For instance, it was recommended to use a pop-up window with the sentence.
5. **Crags' information:** In the system V&C, first, crags are shown in the interface of a map, and second, when climbers choose a certain crag, they can observe additional information about the crag with icons and explanations. Several important aspects have been suggested to be included: *distance of the climbing routes* in meters because sportspeople need to know the length of the climbing rope they should bring with them; the *number of quickdraws* in each route within the crag because athletes need to know the number of quickdraws they should bring to be able to climb a certain route; information about the *type of stations on the top* to understand how they should abseil; *parking spot location* to know where to park. Participants also suggested recommending crags based on several categories: *beautiful views*, crags *located near the lake*, and *cultural places* such as museums that could be visited nearby. Moreover, one should incorporate the reviews of other climbers who visited the crags and climbed the target routes into a climbing RS to improve confidence and trust.

## 5. Discussion and conclusion

In this study, we hypothesized that climbers' previous visits would benefit recommender systems, suggesting outdoor climbing crags: they can be used to model a person's preferences for crags' features and their context. The motivation behind this hypothesis is based on the fact that climbers' visits correlate with crags' features and that users' preferences can be learned from their visits accordingly. Then, we assumed that users with similar tastes would visit similar places, and following this assumption developed a model Visit & Climb to predict the potential climbers' visits to crags. In an offline evaluation scenario, we compared V&C with other RS models (regression-based, matrix factorization, and collaborative filtering) *via* metrics MAP, Recall, and NDCG at top-k levels (k = 1 and 3). The results showed that the V&C provides better recommendations than the baseline model (where the baseline considers past visits by the user as his/her future ones) and as accurate suggestions as the other systems.

To further test the hypothesis, we performed a formative study with 73 climbers, asking them to comment on other existing e-guidebooks and recommender systems for climbing crags. Moreover, we recorded three sessions on how experienced outdoor climbers familiar with web guide tool search for the crags on the web if they plan a climbing vacation. Based on this study, we developed a web interface for Visit & Climb system to facilitate climbers in choosing the suitable climbing rocks in Arco, where the preferences are learned from the users' past visits and can be further adjusted by them with the slider-based preferences elicitation panel. We tested RS with a user-centric evaluation framework in a within-subjects study design: we asked 40 experienced outdoor climbers to try three systems (including V&C) for a hypothetical scenario of their planned climbing trip in Arco. The results of this study showed that the system of Visit & Climb achieves higher metrics for several aspects of recommendations than the other similar systems in this domain.

Overall, recommender systems for outdoor climbing is still a novel area by developing the V&C system; we also wanted to understand whether the users might perceive the slider-based PE panel as a good tool for climbing crag search. The results showed that the slider-based interface provides a high level of information sufficiency caused by transparency. At the same time, for particular crags' features, it is better to adopt a button-based PE and pre-filtering techniques for contextual factor adjustment (some users have found adjusting 33 components exhausting). Some items' characteristics are crucial for choosing crags (tourists traveling with children can only climb in a place suitable for children), while others are unimportant and could be removed. The best approach would be to combine some aspects of Content-Based RS and Visit&Climb RS in the PE interface: buttons for certain crucial features (“*Sun exposure*,” “*Suitable months to climb*,” “*Parking*,” “*Wall steepness*,” “*Type of rock*,” “*Safety*,” “*Rain safe*,” “*Rating*,” and “*Family-friendly*”), and sliders or switchers for some other features (“*Approach time*,” “*The number of climbing routes for fourteen grades/difficulty levels*,” “*Shortest route*,” and “*Longest route*”).

## 6. Limitations

There are certain limitations of the system. First, online evaluation is performed with a small-scale user study (*N* = 40). Second, other exciting web sources could be considered as an alternative to RS V&C, for instance, ukclimbing.com.^17^ Third, during the formative study, we received additional essential comments about the system, which provided an idea of what should be done in future studies. For instance, interface improvement: one professional designer among all participants gave a negative feedback about the interface layout of V&C. It would be interesting to collaborate with a professional designer to develop a new interface and see whether the other metrics, such as overall satisfaction, would improve.

We received another comment about the current recommendations; they need to be better aligned with users' preferences. For instance, users can only visit crags suitable for a season when they plan to see it (summer/winter crags). We assume that the essential improvement can be made by applying contextual pre-filtering. Moreover, incorporating the recent comments from other people who already visited places should be used to better filter crags based on a particular category, e.g., beautiful view, lake location nearby, and problems with equipment within the area.

All those issues are good points to be tackled by researchers who designed a climbing recommender system for the outdoor climbing scenario. We hope this will lead to a more user-friendly interface for sport climbing e-guidebooks and provide more helpful information about rock climbing places, motivating climbers to climb.

## Data availability statement

The datasets presented in this study can be found in online repositories. The names of the repository/repositories and accession number(s) can be found below: https://drive.google.com/drive/folders/1kNXs-vjygKJyYhMdZT-V5Dfhi2jv7LRZ?usp=share_link.

## Ethics statement

The studies involving humans were approved by the Ethics Committee of Free University of Bolzano. The studies were conducted in accordance with the local legislation and institutional requirements. The participants provided their written informed consent to participate in this study.

## Author contributions

II made substantial contributions to conceptualization, investigation, methodology, and analysis and interpretation of data. MW helped in the revision and gave final approval of the version to be published. All authors contributed to the article and approved the submitted version.

## Footnotes

^1^ https://27crags.com/

^2^ https://www.ukclimbing.com/logbook/map

^3^ https://www.8a.nu

^4^ www.mountainproject.com/route-guide

^5^ https://www.thecrag.com/climbing/italy/northern-italy/arco-trento-area/maps#45.9298,10.9032,12.0„auto

^6^ https://www.thecrag.com/en/climbing/italy/massone-policrumble

^7^ https://www.vertical-life.info

^8^ https://www.8a.nu/areas/italy/arco/ascents

^9^ https://scikit-learn.org/stable/modules/generated/sklearn.ensemble.RandomForestRegressor.html

^10^ https://scikit-learn.org/stable/modules/generated/sklearn.linear_model.LinearRegression.html

^11^ https://scikit-learn.org/stable/auto_examples/ensemble/plot_gradient_boosting_regression.html

^12^ https://surprise.readthedocs.io/en/stable/

^13^ https://www.falesia.it

^14^ https://www.google.com

^15^ http://climbing-recommender-visits.site/implicitlogin

^16^ https://www.salewa-cube.com/en

^17^ https://www.ukclimbing.com

## References

[B1] Abdi H. Williams L. J. (2010). Tukey's honestly significant difference (hsd) test. Encycl. Res. Des. 3, 1–5.

[B2] Adomavicius G. Bauman K. Tuzhilin A. Unger M. (2022). Context-Aware Recommender Systems: From Foundations to Recent Developments Context-Aware Recommender Systems. New York, NY: Springer US. 211–250. 10.1007/978-1-0716-2197-4_6

[B3] Bollati I. Zucali M. Giovenco C. Pelfini M. (2014). Geoheritage and sport climbing activities: using the Montestrutto cliff (Austroalpine domain, Western Alps) as an example of scientific and educational representativeness. Italian J. Geosci. 133, 187–199. 10.3301/IJG.2013.24

[B4] Brusilovsky P. de Gemmis M. Felfernig A. Lops P. O'Donovan J. Semeraro G.. (2020). “Interfaces and human decision making for recommender systems,” in Proceedings of the 14th ACM Conference on Recommender Systems, RecSys '20 (New York, NY, USA: Association for Computing Machinery), 613–618. 10.1145/3383313.3411539

[B5] Calbimonte J.-P. Martin S. Calvaresi D. Cotting A. (2021). “A platform for difficulty assessment and recommendation of hiking trails,” in Information and Communication Technologies in Tourism 2021, eds. W. Wörndl, C. Koo, and J. L. Stienmetz (Cham: Springer International Publishing), 109–122. 10.1007/978-3-030-65785-7_9

[B6] Calbimonte J.-P. Martin S. Calvaresi D. Zappelaz N. Cotting A. (2020). “Semantic data models for hiking trail difficulty assessment,” in Information and Communication Technologies in Tourism 2020, eds. J. Neidhardt, and W. Wörndl (Cham: Springer International Publishing), 295–306. 10.1007/978-3-030-36737-4_24

[B7] De Giorgi L. Warasin P. Kaufmann J. Rossi G. Piazzi M. (2021). Red Rocks. Bozen: Alpenverein Südtirol.

[B8] Draper N. (2016). “Climbing grades: Systems and subjectivity,” in The Science of Climbing and Mountaineering (London: Routledge), 227–240.

[B9] Draper N. Canalejo J. C. Fryer S. Dickson T. Winter D. Ellis G.. (2011). Reporting climbing grades and grouping categories for rock climbing. Isokinetics Exer. Sci. 19, 273–280. 10.3233/IES-2011-0424

[B10] Draper N. Giles D. Schöffl V. Konstantin Fuss F. Watts P. Wolf P.. (2015). Comparative grading scales, statistical analyses, climber descriptors and ability grouping: International rock climbing research association position statement. Sports Technol. 8, 88–94. 10.1080/19346182.2015.1107081

[B11] Feely C. Caulfield B. Lawlor A. Smyth B. (2022). “An extended case-based approach to race-time prediction for recreational marathon runners,” in Case-Based Reasoning Research and Development, eds. M. T. Keane, and N. Wiratunga (Cham: Springer International Publishing), 335–349. 10.1007/978-3-031-14923-8_22

[B12] Ginantra N. L. W. S. R. Bhawika G. W. Zamsuri A. Budianto F. Daengs G. A. (2020). “Decision support system in recommending climbing tourism destinations with profile matching method,” in IOP Conference Series: Materials Science and Engineering 1–6.

[B13] Gunawardana A. Shani G. Yogev S. (2022). Evaluating Recommender Systems, 547–601. New York, NY: Springer US. 10.1007/978-1-0716-2197-4_15

[B14] Helle C. Takala T. (2020). Improving user interaction for a content creation web application tool for rock climbing-a case study. Master's thesis, Aalto University, Helsinki, Finland.

[B15] Ivanova I. (2023). Predicting climbers visits with different methods. Available online at: https://github.com/yustiks/predicting-tourists-visits/tree/main/data (accessed August 18, 2023).

[B16] Ivanova I. Andrić M. Ricci F. (2022a). “Content-based recommendations for crags and climbing routes,” in Information and Communication Technologies in Tourism 2022, J. L. Stienmetz, B. Ferrer-Rosell, and D. Massimo (Cham: Springer International Publishing), 369–381. 10.1007/978-3-030-94751-4_33

[B17] Ivanova I. A. Buriro A. Ricci F. (2022b). “Map and content-based climbing recommender system,” in Adjunct Proceedings of the 30th ACM Conference on User Modeling, Adaptation and Personalization, UMAP '22 Adjunct (New York, NY, USA: Association for Computing Machinery), 41–45. 10.1145/3511047.3536416

[B18] Ivanova I. A. Wald M. (2023). “Introducing context in climbing crags recommender system in arco, italy,” in Companion Proceedings of the 28th International Conference on Intelligent User Interfaces, IUI '23 Companion (New York, NY, USA: Association for Computing Machinery), 12–15. 10.1145/3581754.3584120

[B19] Jannach D. Lerche L. Zanker M. (2018). Recommending Based on Implicit Feedback. Cham: Springer International Publishing. 10.1007/978-3-319-90092-6_14

[B20] Knijnenburg B. P. Willemsen M. C. (2009). “Understanding the effect of adaptive preference elicitation methods on user satisfaction of a recommender system,” in Proceedings of the Third ACM Conference on Recommender Systems, RecSys '09 (New York, NY, USA: Association for Computing Machinery), 381–384. 10.1145/1639714.1639793

[B21] Knijnenburg B. P. Willemsen M. C. (2015). Evaluating Recommender Systems with User Experiments. Boston, MA: Springer US. 10.1007/978-1-4899-7637-6_9

[B22] Knijnenburg B. P. Willemsen M. C. Gantner Z. Soncu H. Newell C. (2012). Explaining the user experience of recommender systems. User Model. User-Adapt. Inter. 22, 441–504. 10.1007/s11257-011-9118-4

[B23] Knoch S. Chapko A. Emrich A. Werth D. Loos P. (2012). “A context-aware running route recommender learning from user histories using artificial neural networks,” in 2012 23rd International Workshop on Database and Expert Systems Applications 106–110. 10.1109/DEXA.2012.49

[B24] Lean J. R. (2010). A comparative study of interactive rockclimbing guidebooks and conventional hardcopy guidebooks. Master's thesis, Queensland University of Technology.

[B25] Long J. Jia J. Xu H. (2017). Senserun: Real-time running routes recommendation towards providing pleasant running experiences. Proc. AAAI Conf. Artif. Intell. 31, 5101–5102. 10.1609/aaai.v31i1.10535

[B26] Posti M. Schöning J. Häkkilä J. (2014). “Unexpected journeys with the hobbit: The design and evaluation of an asocial hiking app,” in Proceedings of the 2014 Conference on Designing Interactive Systems, DIS '14 (New York, NY, USA: Association for Computing Machinery), 637–646. 10.1145/2598510.2598592

[B27] Pu P. Chen L. Hu R. (2011). “A user-centric evaluation framework for recommender systems,” in Proceedings of the Fifth ACM Conference on Recommender Systems, RecSys '11 (New York, NY, USA: Association for Computing Machinery), 157–164. 10.1145/2043932.2043962

[B28] Ricci F. Rokach L. Shapira B. (2022). Recommender Systems: Techniques, Applications, and Challenges. New York, NY: Springer US. 10.1007/978-1-0716-2197-4

[B29] Scarff D. (2020). Estimation of climbing route difficulty using whole-history rating. arXiv preprint arXiv:2001.05388.

[B30] Seifert L. Wolf P. Schweizer A. (2016). The Science of Climbing and Mountaineering. New York, NY: Taylor &Francis. 10.4324/9781315682433

[B31] Tintarev N. Masthoff J. (2022). Beyond Explaining Single Item Recommendations. New York, NY: Springer US.

[B32] Vardhan M. Hegde N. Merugu S. Prabhat S. Nathani D. Seneviratne M.. (2022). “Walking with pace - personalized and automated coaching engine,” in Proceedings of the 30th ACM Conference on User Modeling, Adaptation and Personalization, UMAP '22 (New York, NY, USA: Association for Computing Machinery), 57–68. 10.1145/3503252.3531301

[B33] Wilkes M. Janowicz K. (2008). “A graph-based alignment approach to similarity between climbing routes,” in Proceedings of the First International Workshop on Information Semantics and its Implications for Geographic Analysis (ISGA) (Citeseer), 1–6.

